# Constructions of sex and intimacy after cancer: Q methodology study of people with cancer, their partners, and health professionals

**DOI:** 10.1186/1471-2407-13-270

**Published:** 2013-05-31

**Authors:** Janette Perz, Jane M Ussher, Emilee Gilbert

**Affiliations:** 1 Centre for Health Research, University of Western Sydney, Locked Bag 1797, Penrith South, 2751, Australia

**Keywords:** Cancer and sexuality, Q methodology, Intimacy, Quality of life, Communication

## Abstract

**Background:**

The increasing number of individuals living with cancer has led to a focus on the quality of life of survivors, and their families. Sexual wellbeing is a central component of quality of life, with a growing body of research demonstrating the association between cancer and changes to sexuality and intimacy. However, little is known about patient and professional understanding of cancer and sexuality post-cancer. This study was designed to explore the complex perspectives that people with personal and professional experience with cancer hold about sexuality in the context of cancer.

**Methods:**

An interview study using Q methodology was conducted with 44 people with cancer, 35 partners of a person with cancer and 37 health professionals working in oncology. Participants were asked to rank-order 56 statements about sexuality and intimacy after cancer and asked to comment on their rankings in a subsequent semi-structured interview. A by-person factor analysis was performed with factors extracted according to the centroid method with a varimax rotation.

**Results:**

A three-factor solution provided the best conceptual fit for the perspectives regarding intimacy and sexuality post-cancer. Factor 1, entitled “communication – dispelling myths about sex and intimacy” positions communication as central to the acceptance of a range of satisfying sexual and intimate practices post-cancer. Factor 2, “valuing sexuality across the cancer journey,” centres on the theme of normalizing the experience of sex after cancer through the renegotiation of sex and intimacy: the development of alternative sexual practices. Factor 3, “intimacy beyond sex,” presents the view that even though sex may not be wanted, desired, or even possible following cancer, quality of life and relationship satisfaction are achieved through communication and non-genital intimacy.

**Conclusions:**

This study has demonstrated the complexity of perspectives about sexuality and intimacy post cancer, which has practical implications for those working in cancer care and survivorship. Therapists and other health professionals can play an important role in ameliorating concerns surrounding sexual wellbeing after cancer, by opening and facilitating discussion of sexuality and intimacy amongst couples affected by cancer, as well as providing information that normalizes a range of sexual and intimate practices.

## Background

More than 12.7 million cases of cancer are estimated to have occurred worldwide in 2008 [1], with over 100,000 new cases diagnosed in Australia [2]. With survival rates at 5 years currently over 60% [2], increasing numbers of individuals are living with cancer, leading to a focus on the quality of life of survivors, and their families. Sexual wellbeing is a central component of quality of life [3], and there is a growing body of research demonstrating the association between cancer and changes to sexuality and intimacy [4-7]. For example, women with cancer have reported reductions in sexual interest and vaginal lubrication [8], early menopause [9], and dyspareunia [10-12], associated with diminished body image [13,14] and sexual confidence [8,15], as well as dissatisfaction with intimate relationships [16]. Similarly, men with cancer have reported changes in ejaculatory capacity, erectile potential [17], orgasm, and urinary incontinence [18], leading to a diminished sense of manliness, self worth, and sexual confidence [18,19]. These changes can lead to significant distress, which in some instances can be experienced as the most difficult aspect of life following cancer [20].

It is now widely recognised that obtaining information about sexual changes after cancer is associated with positive psychological and sexual adjustment [15,21], leading to the recommendation that education and training of health professionals working in oncology should include a focus on sexual issues [22,23]. However, in a review of the construction of patient sexuality in the cancer research literature, it was reported that the focus is predominantly on the relationship between cancer treatments and sexual dysfunction, with ‘sex’ conceptualised as penis-vagina intercourse [24], described elsewhere as the “coital imperative” [25], p. 44, [26], p. 229. This serves to position sexuality within a narrow hetero-normative framework [27], negating intimacy and non-coital sexual practices, as well as the complex social, psychological and relational context within which sexuality and intimacy are experienced [24,28].

These same narrow constructions were identified by Hordern and Street (2007a) in interviews with Australian health professionals working in cancer care, where sex was positioned as secondary to survival, or as a difficult topic to discuss. In contrast, patients and partners report that they want information and support about sexual changes after cancer [10], with many individuals adopting a “plastic” view of sexuality [23], wherein the meaning and practice of sex and intimacy can be renegotiated after cancer to include non-coital sexual practices [29]. Given these “mismatched expectations” [23], p. 224 it is not surprising to find that many people with cancer and their partners report a high level of dissatisfaction with health professional communication about sex and intimacy [5,10], as well as reporting that their sexual information and support needs are not being met [30].

Since Hordern and Street’s research was conducted, there has been a proliferation of research documenting changes to sexuality after cancer [6,7,31], leading to renewed emphasis on health professional communication and support [22,24,32,33]. This begs the question: does the “mismatch” between health professional and patient perspectives on sexuality and cancer remain? The aim of the present study is to address this question, by examining the perspectives of health professionals alongside those of people with cancer and their partners.

Previous research on cancer and sexuality has focused largely on cancers that directly affect the sexual or reproductive organs, such as prostate, testicular, breast and gynaecological cancer [11,12,34,35]. However, there is evidence that cancer also has an impact on the sexual wellbeing of people with non-reproductive cancers, such as lung and colorectal cancer e.g. [36,37]. Equally, while there is evidence that changes to sexuality are experienced by the intimate partners of people with both reproductive and non-reproductive cancers [5,38], partners are rarely included in research on cancer and sexuality. The aim of this study is to examine constructions of sexuality adopted by a range of health professionals working in cancer care, as well as those of patients and their partners, across a range of cancer types, using Q methodology.

### Q methodology

Q methodology was developed by William Stephenson in the 1930s as a technique for “revealing the subjectivity involved in any situation” [39], p. 561. Q methodology provides a means for sampling subjective viewpoints, and can be used to identify patterns, including areas of overlap or difference, across various perspectives on a given topic [40,41]. The method is described as “qualiquantilogical” combining elements from qualitative and quantitative research traditions [42]. The data consists of participant’s constructions of a given topic, obtained by ranking a set of pre-defined items according to his or her perspective and experience. The resulting factors from the subsequent factor analysis indicate constructions of subjectivity that exist within the rankings [39]. The factor analysis performed in Q methodology is used to identify associations between patterns expressed by participants, a procedural inversion to conventional factor analysis that is used to identify associations between variables [43]. As such, the focus in Q methodology is not the ‘constructors’ (the participants), but the ‘constructions’ themselves [44].

Q methodology has been described as a more robust technique than alternative methods for the measurement of subjective opinion, and has been recommended in the study of attitudes within the health field [45]. The method has been applied to the study of a wide range of topics in health and medicine including an understanding of irritable bowel syndrome [46], needs appraisals in neurodegenerative disorders [47], beliefs about the sexuality of the intellectually disabled [48], perceptions of childhood obesity [40], and experiences of postnatal perineal morbidity [49]. With the exception of one study exploring perceptions of fatigue amongst adolescents with cancer [50], the potential of this approach has not been utilised in the cancer field. This paper presents the results from a study using Q methodology, which aimed to produce a classification of constructions of sexuality in the context of cancer from various personal and professional perspectives. Consistent with the principle of “finite diversity” [44], that limited variability exists among the accounts that people construct, it was expected that several ordered patterns of shared understanding would be identified, according to broader social and cultural experiences and constructions.

## Methods

Q method begins with a Q set or sample which is composed of statements, rather than participants, reflecting Q’s focus with discourses and how discourses variously come together [44]. A participant group (P set) is selected from as many of the obviously pertinent demographic groups as possible for the topic [51]. Unlike traditional rating scales that work with absolute responses to statements, Q sorting involves the ranking of statements with relative agreement or disagreement, where statements only become meaningful in relation to position of other statements [43]. In Q analysis, an inverted factor analytic procedure, overall configurations of statements, or factors, are produced that are shared by the participants who load onto to that factor, a procedure that detects associations between patterns expressed by persons [43]. These features of the Q method are described below for the current study, with further description of the principles and method of Q methodology described elsewhere [51].

### Item sampling – development of the Q set

Q methodology begins with the development of a set of items that is a broadly representative sample of perspectives and viewpoints across a given topic. Although there is no single method for generating a Q set, two characteristics mark an effective Q set – coverage and balance [51]. For coverage, “the items must cover all the ground within the relevant conceptual space” (p. 58), whereas, balance is achieved by ensuring that the Q set is not biased towards a particular viewpoint. In the present study, a sample pool of items was elicited from a number of naturalistic sources [52,53] in order to capture real-world examples of discourses around the topic of sexuality in the context of cancer. This included an extensive review of the academic literature on cancer and sexuality, media and popular culture materials, and pilot interviews with people with cancer, their partners, and health care professionals. Structured sampling was performed where item content was organised under themes emergent in the data sources [52]. In this way, 135 pool items were structured around 7 themes: sexual attractiveness and body image; intimacy and love; ‘appropriate’ sexual behaviour; physiological effects of cancer and cancer treatments; couple communication; role of the health professional; and renegotiating sexual practices. Statements were refined into a final Q set of 56 items by removing repetition, duplication and ambiguity. Content and face-validity were ensured by subjecting the final set of items to independent assessment and review by oncology clinicians and consumer representatives drawn from an advisory committee established for the research project [40]. Finally, the statements were edited and reworded to ensure that each expressed a distinct perspective on sexuality within the context of cancer, randomly numbered and each one printed on a separate card for ease of sorting.

### Recruitment and participants – the P set

This study was part of a larger mixed-methods cross-sectional project examining multiple perspectives on sexuality and intimacy post-cancer in Australia. The larger project from which participants were drawn was advertised via cancer organisations, websites and newsletters, as well as through media releases, cancer support groups, and cancer care clinics nationally. Six hundred and ninety-eight people with cancer and 175 partners of a person with cancer completed an online or postal questionnaire examining their experiences of intimacy and sexuality post-cancer. Of the 873 survey respondents, 274 responded positively to an invitation to take part in a Q sort activity and semi-structured individual interviews. We selected 79 for interview, representing a cross section of cancer types and stages, gender, and sexual orientation, reflecting the larger study population. In addition, 37 health care professionals working in oncology were recruited nationally via professional bodies, networks and conferences, and were also invited to take-part in a Q sort activity and semi-structured individual interviews regarding their experiences of sexuality and intimacy within the context of cancer care. Ethical approval was granted from relevant human research ethics committees, including the University of Western Sydney, and Sydney West and South Eastern Sydney Illawarra area health services. As per the approved ethical protocol for the study, written informed consent was obtained from all participants.

Participant selection was guided by the aim to maximise the possibility that a variety of perspectives could be expressed [42]. Participant selection was strategic to ensure that participants likely to express interesting or pivotal points of view with respect to their experience with cancer were included [51]. The P set, participant group, consisted of 116 adults ranging in age from 20 to 77 years (*M*_age_ = 52; *SD* = 13) consisting of 44 people with cancer (23 women, 21 men, *M*_age_ = 54.2, *SD* = 14.4), 35 partners of a person with cancer (18 women, 17 men, *M*_age_ = 54.6, *SD* = 12.8), and 37 health professionals (32 women, 5 men, *M*_age_ = 46.9, *SD* = 10.1). For the people with cancer and partners, 86% were currently in a relationship, 87% identified as heterosexual, and 91% nominated Anglo-Australian as the cultural group with which they identified. Further diversity was achieved by stratified sampling within participant sub-groupings for variables of theoretical significance. In this study, 42 people with cancer and partners (53%) were associated with a reproductive cancer (e.g. prostrate, breast, gynecological) and 37 (47%) were associated with a non-reproductive cancer type (e.g. lung, bowel, pancreatic, mesothelioma). Seventy-one participants reported one cancer diagnosis (89.9%) and 8 reported multiple cancer diagnoses (10.1%), with 69% being in remission, with treatment completed, 28% in active treatment, and 3% bereaved partners. Research has identified a role for a range of health professionals in oncology where discussions around cancer and sexuality may occur [54]. To capture viewpoints from a range of professional groups, 9 doctors, 11 nurses, 10 psychologists and 7 social workers completed the Q sort.

### Procedure – Q sorting

**Figure 1:**
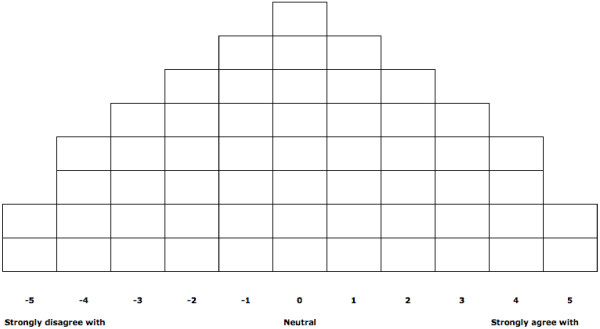
Participant response grid (Q grid).

A pack was sent to each participant that included a participant information sheet and consent form explaining the study and their involvement, instructions for completion, 56 statement cards, response grid (Q grid) in the shape of a quasi-normal distribution, and a reply-paid envelope for the return of materials. Participants were asked to arrange the statements about cancer and sexuality into the Q grid (Figure 1) according to those that they least agree with (-5) to those that they most agree with (+5). The instructions suggested that participants initially sort into three piles (agree, disagree, and neutral) and then continue the sorting until all statements were assigned using the Q grid, with only one statement placed in each cell. In this ‘forced’ sorting process, participants’ subjective neutrality is indicated by statements placed nearer the center of the grid [53]. Prior to recording their statement placements, participants were encouraged to review the final array and make any changes were they felt appropriate. At the commencement of the semi-structured interview, participants were asked to comment on their positioning of statements, especially those items placed at the poles and any other statements of interest to the participant. Interviews were subsequently transcribed verbatim to allow for participant comments to be used in the interpretation of the Q analysis.

### Q analysis and factor interpretation

In Q analysis, a by-person factor analysis is performed to identify perspectives or viewpoints subscribed to by a number of participants. That is, the factor analysis groups together participants who have sorted the Q sample similarly [55]. Completed participant response grids were collated with factors extracted according to the centroid method and then rotated using PCQ for Windows [56]. This extraction technique maximizes the total explained variance with each successive factor extracted. The openness and permissiveness of this technique allows the researcher to examine the data set from a variety of perspectives [51]. The emergent factor solution was subjected to a varimax rotation, which has been demonstrated statistically and theoretically to be a sound method of factor rotation [42]. In Q analysis, a varimax rotation positions factors so that the overall rotated solution accounts for as much of the explained variance as possible. This is achieved by ensuring that each Q sort (participant) has a high factor loading on only one factor, an analytic technique that can reveal the majority viewpoints of the sample [51]. In line with the aim of identifying shared perspectives, interpretable Q factors were selected if they met the stringent criteria of eigenvalues greater than one and at least two factor exemplars, that is, Q sorts (participant responses) loading significantly upon one factor alone [42]. The best conceptual fit for this study of the perspectives of people with cancer, their partners, and health professionals regarding intimacy and sexuality post-cancer was a three-factor solution.

Factor arrays, the merged average of the Q sorts, provide a conceptual representation of that factor. The values represent the “archetypal” pattern sort for participants who define each factor [57]. Factor interpretation was based upon a thematic reading of statements and their position in the context of all other statements in the final factor arrays. Differences between factors were articulated by examining distinguishing statements (statements with statistically different factor scores across factor arrays). Consensus statements (statements that do not distinguish between any of the significant factors) were also examined to identify similarities between the factors. In generating the nuanced meaning of each factor, participant interview comments, both general and those related to specific statements, and factor exemplar socio-demographic profiles, were used.

## Results

Centroid analysis produced 7 factors accounting for 68% of total variance. Following varimax rotation, six factors had eigenvalues greater than 1.0, with the first three factors additionally defined by factor exemplars with factor loadings greater than 0.5. As an estimate of the construct validity of each factor, composite reliability coefficients (r_c_) greatly exceeded the minimum acceptable value of > 0.7. Table 1 shows the factor characteristics for these factors. Table 2 presents the socio-demographic and clinical profile for the Q sorts defining each significant factor. Factors scores of each statement across all three factors are displayed in Table 3. Statements discriminating among the factors are highlighted. In the factor descriptions, bracketed notations represent statement rankings within factor arrays, in that (13: +5) indicates that statement 13 is ranked in the +5 (most agree with) position.

**Table 1:** Q factor characteristics

	**Factor**						
Characteristic	1	2	3	4	5	6	7
Number of defining sorts	31	24	6	0	0	1	1
Composite reliability	0.99	0.99	0.96	0	0	0.80	0.80
Eigenvalue	28.15	26.53	12.93	4.94	0.13	4.00	2.33
% of explained variance	24.27	22.87	11.14	4.26	0.12	3.44	2.01

**Table 2:** Socio-demographic and clinical information for Q sorts (participants) defining each factor

**Variable**	**Factor 1 (*n*=31)**	**Factor 2 (*n*=24)**	**Factor 3 (*n*=6)**
	**(Count, %)**	**(Count, %)**	**(Count, %)**
Group			
Person with cancer	6 (19.4)	12 (50)	3 (50)
Partner of a person with cancer	2 (6.5)	12 (50)	3 (50)
Health care professional	23 (74.1)	-	-
*Doctor/Surgeon*	*6 (19.4)*		
*Nurse*	*5 (16.1)*		
*Allied health*	*12 (38.7)*		
Gender			
Female	27 (87.1)	9 (37.5)	3 (50)
Male	4 (12.9)	15 (62.5)	3 (50)
Age (*Mean, S.D.)*	43.8 (12.4)	57.7 (11.4)	51.7 (10.5)
Type of cancer*			
Reproductive	2 (25)#	15 (62.5)	2 (33.3)
Non-reproductive	6 (75)#	9 (37.5)	4 (66.7)
Relationship status*			
Partnered	7 (87.5)#	22 (91.6)	4 (66.7)
Not partnered	1 (12.5)#	1 (4.2)	2 (33.3)
Partner deceased	-	1 (4.2)	-
Sexual orientation*			
Heterosexual	7 (87.5)#	20 (83.3)	5 (83.3)
Non-heterosexual	1 (12.5)#	4 (16.6)	1 (16.7)

* Indicates that the variable is calculated for participants who are a person with cancer or a partner of a person with cancer only.

# Percentage calculated only for defining participants on Factor 1 who are a person with cancer or a partner of a person with cancer.

**Table 3:** Q-set statements and factor array

		**Factor**		
Item		1	2	3
1	People with cancer are sexually unattractive	−3	−3	**−3**
2	People with cancer don’t have any sexual desires	−3	−3	0
3	Partners of people with cancer can lose their interest in sex	+2	0	+1
4	People with cancer do not have the energy for sex	+1	−1	+2
5	People with cancer should focus on recovery and survival rather than on their sexual relationship	−2	0	+2
6	The sexual relationship is the last thing on a couple’s mind when one of them has cancer	−1	−1	+2
7	Cancer doesn’t remove the sense of duty that couples share in their sexual relationship	+1	+1	−1
8	Fear of hurting someone with cancer during sex stops partners from having a sexual relationship with them	+2	0	+1
9	A person would feel disgusted with themselves for having sex with a person with cancer	−2	−4	−3
10	Once someone is seen as a “patient”, they can’t be seen as a sexual partner	−3	−4	−3
11	There isn’t anything sexy about caring for someone with cancer	−1	−1	+1
12	People should always make time for sex even if they have cancer	−1	+2	−1
13	Intimacy means more than just sex	**+5**	**+4**	**+5**
14	Cancer can bring couples closer in their intimate relationship	+2	**+3**	+3
15	Just being held may be all that the person with cancer wants	**+4**	+3	**+5**
16	Cancer results in greater affection between partners	0	+2	+1
17	When a person is diagnosed with cancer, sex becomes taboo	0	**−5**	−3
18	Partners of people with cancer would feel guilty if they initiate sex	+1	−3	+2
19	Partners of people with cancer should feel guilty for having sexual needs	−3	−3	−2
20	Sex should be a low priority in the context of cancer	−2	−2	+2
21	Only cancer involving “sexual” body parts (e.g. testicles, breast, prostate, cervix) affects a sexual relationship	−3	−1	−3
22	Sex can no longer be spontaneous after the onset of cancer	−2	−2	0
23	Partners who care for a person with cancer are too tired for sex	0	−2	−1
24	Having sex with someone with cancer may hurt them	−1	0	0
25	It would be embarrassing for a person with cancer to ask their partner to have sex differently	0	−2	−2
26	A partner wouldn’t bring up the idea of using a sex toy for fear of hurting the feelings of the person with cancer	0	−1	0
27	A partner should be able to talk about their sexual needs to a person with cancer	+3	**+4**	0
28	A person with cancer should be able to talk about their sexual needs to their partner	+3	**+5**	+3
29	Open communication between people with cancer and their partner is important to a satisfying sexual relationship	**+4**	**+5**	**+4**
30	It’s important that health care professionals discuss the affect of cancer on the sexual relationship with partners of people with cancer	**+4**	+2	0
31	The sexual relationships of people with cancer are too personal an issue for health care professionals to discuss	**−4**	−2	−2
32	The sexual relationship between a couple is a private matter	0	+1	+3
33	Health care professionals should discuss with people with cancer how cancer affects their sexual relationship	**+4**	+2	0
34	Sex therapy is as important as other therapies a person receives for cancer	+2	+2	2
35	If people with cancer and their partner cannot have sexual intercourse, they find other ways to be sexually intimate	+3	+3	+3
36	If people with cancer and their partner cannot have a sexual relationship, their relationship is over	**−4**	**−4**	**−4**
37	Real sex is penetrative sexual intercourse	**−4**	−1	**−4**
38	It is okay for people with cancer and their partner to masturbate for sexual pleasure	+3	**+4**	+3
39	Sexual aides keep a sexual relationship alive for people with cancer and their partner	+1	+1	2
40	Physical changes that result from cancer would make you feel less confident about sex	+2	+1	+1
41	If a man with cancer can’t get an erection, there is no point in being intimate	**−4**	**−4**	**−4**
42	If a woman with cancer can’t get aroused, there is no point in being intimate	−2	−2	−1
43	Once you’ve lost control of your bodily functions, it’s hard to think about sex	+2	+1	**+4**
44	Partners would feel rejected if the person with cancer doesn’t want to have sex	+1	0	−1
45	People with cancer should not be burdened by the sexual needs of their partner	−1	0	+2
46	It is depression that ends the sexual relationship in couples dealing with cancer	−1	+1	−1
47	The sexual relationship goes up and down with the rollercoaster of emotions experienced during cancer	+3	+2	**+4**
48	Sex is good for dealing with the anxiety that comes with cancer	+1	+3	−1
49	You shouldn’t have sex with someone while they are receiving chemotherapy	−2	−1	0
50	If you have sex with someone with cancer, there is a real risk of catching it	**−5**	**−5**	**−5**
51	You can’t spread cancer through sex	**+5**	+3	**+4**
52	In the early stages of cancer, it’s important to maintain a sexual relationship to keep things normal	0	**+4**	−2
53	A sexual relationship is key to maintaining a good quality of life during the advanced stages of cancer	−1	+1	**−4**
54	Sex can make cancer worse	**−5**	−3	**−5**
55	Sex can’t be pleasurable if you’re experiencing pain	+1	0	+1
56	Cancer makes parts of the body off limits	0	0	+1

Bolded scores are for statements with normalized factor scores ≥±1.4 indicating the most powerful exemplars for each factor.

### Factor 1: “communication - dispelling myths about sex and intimacy”

Factor 1 accounted for 24% of total variance with the Q sorts of 31 participants defining this factor. Of these factor exemplars, 74% were health professionals, 87% female, and the mean age was 43.8 years. For those defining participants who were people with cancer or partners, 75% had experience with a non-reproductive cancer and 87% were in partnered, heterosexual relationships.

The Factor 1 position is oriented around the importance of communication about sex and intimacy in the context of cancer, in particular, the role of health professional communication with patients and their partners. Defining participants strongly endorsed health professionals discussing the effect of cancer on sexual relationships with people with cancer (33: +4) and the partners of people with cancer (30: +4), and rejected the notion that the sexual relationships of people with cancer are too personal an issue for health professionals to discuss (31: -4). This is illustrated by the following participant comments: “If we’re not educating our patients about sex and sexuality, who else is going to do it” (nurse, female, 49yrs); “patients are reluctant to address it and I feel that I’m fairly proactive. And I hope that I tailor things to the client” (psychologist, female, 55yrs). This endorsement of the role of health professionals discussing sexual issues distinguished defining participants for this factor from those in Factors 2 and 3, with the latter not as negative in their responses to the notion that the sexual relationships of people with cancer are too personal an issue for health professionals to discuss (31: -2), and more likely to agree with the suggestion that the sexual relationship between a couple is a private matter (32: +1, +3).

In addition to supporting discussions with health professionals, defining participants for Factor 1 also strongly agree that open communication between people with cancer and their partners is important to a satisfying sexual relationship (29: +4), illustrated by the comment of one participant that successful sexual renegotiation, the development of alternative sexual practices when coital sex was difficult or painful [10,29], resulted from couples “being able to communication” (doctor, male, 35yrs). Another participant said: “open communication is an important part of any sexual relationship, regardless of cancer” (social worker, female, 31yrs). These participants share a broad understanding of what sex is, rejecting the view that ‘real sex’ is penetrative intercourse (37: -4), and affirming that intimacy means more than just sex (13: +5): “A lot of women post mastectomy just find that just lying with their partner or getting a massage from their partner is as much intimacy as they can have and all that they require at that time” (nurse, female, 47 years); “women shouldn’t feel that because they’ve got a vulval cancer for example you know – had their clitoris removed and their labia removed or whatever; they shouldn’t feel that there’s not anything that they can do sexually. They can do all sorts of things” (psychologist, female, 63yrs). In the context of cancer, holding the person with cancer is supported as an intimate practice (15: +4), and the notion that a man’s inability to have an erection means that there is no point in being intimate (41: -4) was rejected: “Men need to be cuddled and just because he can’t get an erection does not mean to say that he doesn’t want to be cuddled or loved” (nurse, female, 58yrs).

In the case where people with cancer and their partners no longer have a sexual relationship, these participants do not accept that this means the couple’s relationship is over (36: -4). Contagion notions about sex and cancer are rebuffed, with strong support for the assertion that you cannot spread cancer through sex (51: +5) and disagreement with predictions that if you have sex with a person with cancer there is a real risk of catching it (50: -5).

### Factor 2: “valuing sexuality across the cancer journey”

Factor 2 has 24 defining participants and accounts for 23% of the study variance. All significantly loading participants were people with cancer, or the partners of people with cancer, 50% respectively. Over 60% of the defining participants were men and the mean age for this group was 57.7 years. The majority of these participants had experience with a reproductive type of cancer (63%). Ninety-two per cent of defining participants for this factor were currently in a relationship, with 83% identifying as heterosexual.

The Factor 2 perspective is focused upon acknowledging the importance of sexuality in maintaining quality of life and supporting the renegotiation of sex and intimacy post-cancer. Open couple communication is central in this perspective with defining participants in strong agreement that a person with cancer (28: +5), and a partner of a person with cancer (27: +4), need to be able to talk about their sexual needs, and recognise the importance of open communication to a satisfying sexual relationship (29: +5). As one participant commented: “to make it work it needs to be talked about between the partners … we’ve found our sexual relationship has improved since the prostate cancer, because it opened up issues that we hadn’t talked about before” (partner, prostate cancer, female, 65yrs). In contrast, these participants reject the suggestion that when a person is diagnosed with cancer, sex becomes taboo (17: -5).

The normalising of sexuality within the context of cancer can be seen in defining participants disagreeing with the claim that once someone is seen as a ‘patient’, they cannot be seen as a sexual partner (10: -4), and the distancing of themselves from statements assigning feelings of guilt to partners of a person with cancer for having sexual needs (19: -3). As two participants commented: “I think just making the effort to prove to them that they’re still a beautiful person and not just, hair (loss) doesn’t make you any different”(partner, breast cancer, male, 39yrs); “If you are really in love with somebody, cancer is something which doesn’t affect in any way of how you feel about that person. It’s just a part of life, it’s something you have to deal with”(partner, breast cancer, male, 50yrs). Feelings of disgust towards those who have sex with a person with cancer (9: -4), and the notion that you can catch cancer by having sex with someone with cancer (50: -5) were also rejected, participants describing the latter as “ridiculous”, “just crazy”, or “that’s nonsense”.

Acceptance of a broad range of sexual and intimate practices was evident, with shared agreement amongst defining participants that intimacy means more than just sex (13: +4), that mutual masturbation for sexual pleasure is okay (38: +4), describing engaging in “outercourse”, “oral sex”, and the use of sex toys, such as vibrators. Defining participants disagreed with the claim that if a man cannot have an erection, there is no point in being intimate (41: -4). As one participant told us: “That whole thing about sex starts when the guy gets an erection and ends when he’s had an orgasm and that’s it … I reject that so deeply it’s not funny” (breast cancer, female, 48yrs). Another said: “I can’t get an erection, but I think we have, I would rate it as nearly as good a sex life as before the operation”(prostate cancer, male, 68yrs).

The shared view amongst these defining participants is that cancer can bring couples closer in their intimate relationships (14: +3), illustrated by the comment: “Sex enhances the relationship and if your relationship is enhanced then obviously you’re going to feel better and it’ll help you have the positive thoughts that I think are important in dealing with cancer” (partner, prostate cancer, female, 56yrs). The positioning of sexuality as important to retaining quality of life across the cancer journey was also a distinguishing feature of this factor. Factor proponents strongly agreed that in order to keep things normal, maintaining a sexual relationship was important in the early stages of cancer (52: +4), reflected in the comment: “when we got the phone call that he had a life threatening illness, it didn t take us very long to end up back into having sex. I think that’s the first thing we did when we saw each other as a way of coping” (partner, leukaemia, female, 31yrs). Defining participants also agreed that sex was the key to maintaining a good quality of life during the advanced stages of cancer (53: +1), supporting the stance that people with cancer should always make time for sex (12: +2): “A lot of cancer is stress related so there’s no better way to relieve stress” (partner, breast cancer, male, 39yrs); “if you’ve got a sexual relationship I think it’s important to, to try and keep that” (bowel cancer, male, 68yrs); it’s just a matter of keeping normality in life really” (partner, melanoma, female, 69yrs). In contrast, defining participants for Factors 1 and 3 disagreed with the views expressed in these statements.

### Factor 3: “intimacy beyond sex”

Accounting for 11% of the study variance, Factor 3 is defined by 6 participants. With a mean age of 51.7 years, 50% of defining participants were men with cancer, the remaining 50% were women partners of a person with cancer. The majority of these participants have had experience with a non-reproductive type of cancer (4 of 6), were currently in a relationship (4 of 6) and 5 of the 6 identified as heterosexual.

The perspective highlighted by Factor 3 stresses the importance of intimacy in relationships post cancer. Defining participants strongly endorsed a broad conception of sexual intimacy, identifying with statements that intimacy means more than sex (13: +5), and that couples can find ways other than sexual intercourse to be intimate (35: +3), including the person with cancer being held (15: +5). This was reflected in the following interview comments: “We explore other things like touching and, and talking about it” (partner, non-Hodgkin lymphoma, male, 35yrs); “those long lingering hugs, I think were saying to each other, ‘Well you know sure, we can’t do the great physical things that we used to do but this is *really* as good a substitute at this point in life” (partner, melanoma, male, 67yrs); “sex is very low priority and not what I wanted but that doesn’t mean you don’t need affection. Just being held is very comforting and good” (lymphoma, female, 57yrs). This emphasis on intimacy is in contrast to the notion that penetrative sex is real sex (37: -4), and that for a man an erection is required for displays of intimacy (41: -4).

A distinguishing feature of this perspective was that sex and the sexual relationship after cancer were not seen as critical to the maintenance of a good quality of life (53: -4), the sustainability of relationships (36: -4), and may not even be a consideration when the effects of cancer result in a loss of control of bodily functions (43: +4). These constructions are illustrated by the comments: “I think if the relationship was over because of the lack of sex then it wasn’t a relationship anyhow” (lymphoma, female, 57yrs); “the last thing on my mind was sex [chuckles] and I was just simply making sure my wife’s final days were as pain free and as loving as I could” (partner, melanoma, male, 67yrs).

The proposition that people with cancer may not have the energy for sex (4: +2), and that people with cancer should focus on recovery and survival rather than on their sexual relationship (5: +2), distinguished this factor from the views expressing the normalisation of sex and cancer seen in Factors 1 and 2. As one participant told us about his wife: “She was in a lot of pain, a lot of physical discomfort with that pain which medication couldn’t cure, if she was to remain conscious. And so I think at that stage a sexual relationship is the last thing on a couple’s mind” (partner, melanoma, male, 67yrs).

Defining participants for Factor 3 were in strong agreement that cancer and its treatments can impact upon the sexual relationship, reflected in the description of the sexual relationship going up and down with the rollercoaster of emotions experienced during cancer (47: +4) and highlighting the importance of open communication in a satisfying sexual relationship (29: +4). Consistent with Factors 1 and 2, proponents of this factor did not accept negative health outcomes attributed to sex during cancer such as catching cancer from having sex with someone who has cancer (50: -5) and the suggestion that sex can make cancer worse (54: -5).

### Consensus statements

Consensus was apparent for 4 statements that did not distinguish between any pair of factors. Defining participants across factors strongly disagreed that if the sexual relationship is over, the relationship for people with cancer and their partners is over (36; -4) and that if a man with cancer cannot get an erection, there is no point in being intimate (41: -4). Consensus was also found around de-stigmatizing cancer and sexuality, with rejection of the view that once seen as a “patient”, a person could not been seen as a sexual partner (10: -3 to -4), and a generally neutral endorsement of the notion that partners would feel rejected if the person with cancer did not want to have sex (44: +1 to -1).

## Discussion

This study was designed to explore the complex constructions that people with personal and professional experience hold about sexuality and intimacy in the context of cancer, with the aim of uncovering discrepancies and commonalities between professional and patient or partner perspectives. In line with the expectations of finite diversity [42], three distinct perspectives were identified reflecting limited variability in the ways in which sexuality is constructed post-cancer. These three accounts represent shared viewpoints about the importance of sexuality to quality of life and relationships, open communication, the role of health professionals, and dispelling falsehoods and misinformation about sexuality and cancer. However, the factors do vary in their evaluation of the importance, and priority of these concerns. Careful consideration of the statements endorsed within each factor along with the socio-demographic characteristics of participants who defined these factors and their interview comments, allowed for a more nuanced interpretation of the salient beliefs represented in these distinct viewpoints.

The first factor perspective, entitled “communication – dispelling myths about sexuality and cancer” positions communication as central to the acceptance of a range of satisfying sexual and intimate practices post-cancer. This viewpoint emphasizes the active involvement of health professionals in discussing the effects of cancer upon sexuality with people with cancer and their partners, as is emphasised in the PLISSIT [58] and BETTER [24] models of health professional communication. This viewpoint also endorsed open communication amongst couples and rejected coital imperative constructions of sex.

For Factor 1, three-quarters of the defining participants were health care professionals with an average of nine years working in oncology. The perspectives evident in this factor contrast with those reported by Hordern and Street (2007a). This could be interpreted as evidence that recent health professional education and training about the importance of the discussion of sex in the context of cancer has had a positive impact. In the interviews which followed the Q sorts, all participants loading on this factor gave accounts of discussing sex and intimacy with many of their patients. However, sexuality was acknowledged to be a difficult subject to address which was often avoided because of patient, health professional, or situational factors [59]. This suggests that the perspectives provided in this study could reflect health professionals adopting a ‘best-practice’ position in relation to the discussion of sex in the context of cancer, knowing that this was the focus of the research study. In this vein, one of the doctors told us:

“Health care professionals should discuss with people with cancer how cancer affects their sexual relations – well I know that that is something that we should do and I’ve put that down as strongly agree. I think not every health professional is comfortable with that and it’s being comfortable with your own (sexuality), being comfortable with your communication skills, being comfortable with the people that you’re sitting with and being comfortable with your role” (female, 45yrs).

Another doctor told us: “what we think we should do and what we actually do are different things often” (male, 35yrs). This suggests that education of health professionals about the importance of sexuality after cancer may not be sufficient to change practice. Attention needs to be paid to the barriers that prevent health professional discussion of sexual matters. Equally, as all of the participants in the present study were volunteers, and a significant number of the participants who loaded on this factor were allied health professionals, it cannot be concluded that a proactive perspective about health professional communication is widely shared; further research with a randomly selected group of health professionals working in oncology is necessary to determine the representative nature of these views.

The second factor, “valuing sexuality across the cancer journey,” centres on the theme of normalizing the experience of sex after cancer through the renegotiation of sex and intimacy, suggesting a plastic model of sexuality was adopted, associated with rejection of the coital imperative [28]. Open communication and discussion are emphasised as important in maintaining relationships, sexuality, and quality of life, but in contrast to Factor 1, it is communication within the couple dyad that is highlighted rather than communication with health care professionals. This supports recent research which reported that couple communication about sex was a priority for women with breast cancer, and was valued more highly than communication with health professionals [10].

For Factor 2, three-fifths of defining participants were men, with the same proportion reporting an experience with a reproductive cancer. Previous research has reported that a majority of men with prostate and testicular cancer seek to maintain coital sexual activity after cancer, through medical assistance, or the use of sexual aids [17,60,61]. However, the finding that defining participants in Factor 2 valued sexual renegotiation, supports previous accounts of men “working around the loss” when dealing with sexual changes after prostate cancer, expressing intimacy through oral sex and touch, rather than penetration [62]: 312, [63], and partners renegotiating sex after cancer through exploration of genital and non-genital intimacy [29].

The third factor perspective, “intimacy beyond sex,” presents the view that even though sex may not be wanted, desired, or even possible following cancer, quality of life and relationship satisfaction are achieved through communication and non-genital intimacy. Similar to Factor 2, but in contrast to Factor 1, communication within the couple dyad is emphasised. This perspective presented the most inclusive conceptualisation of non-coital or non-genital practices as expressions of intimacy, conceptualising sex as a private matter for couples, and not seeing a sexual relationship as central to quality of life in advanced stages of cancer. This stands in contrast to previous reports of cessation of sex and intimacy when coital sex is not possible after cancer [5], and reports of high levels of distress associated with sexual changes after cancer [12].

Factor 3 was equally defined by men with cancer and women partners of a person with cancer, the latter being most strongly represented in this factor. This suggests that performance of coital sex does not have to be central to relationship satisfaction or gender identity, and that sex is not always a major concern for couples with cancer. Contrary to phallocentric models of masculinity [64], it also confirms previous findings that some men can feel “bloody rapt” (very relieved) [65]: 59] about the possibility of sex without penetration as it serves to reduce performance pressure and offers escape from the “rigidity of what it means to be a man” [17: 1605]. Equally, the high representation of women partners in this factor supports previous research which reported that a significant proportion of women partners of men with prostate cancer did not encourage help-seeking for a sexual problem, with many women resigned to, or accepting of, sexual changes [60]. Sex which is less focused on penetration, and more focused on other forms of intimacy, has also been reported to be preferred by many midlife and older women [66-68], with hugging, kissing, touching [69], as well as relationship closeness [70], being included in definitions of sex. Cancer may serve to legitimate such women resisting the coital imperative, and exploring non-coital, non-genital intimacy.

A few items were viewed in a broadly similar way across the factors. This common perspective was concerned with de-stigmatizing cancer and sexuality and challenging dominant discourses around the centrality of penetrative sex. This suggests that acceptance of the importance of sex and intimacy for people with cancer and their partners, as well as rejection of the coital imperative, particularly in cases where coital sex is difficult or no longer possible, was a construction shared universally by participants. This suggests that research and interventions focused on the experience of sex in the context of cancer which concentrate on coital sex are not encapsulating the broad conceptualisation of sex and intimacy held by people with cancer and their partners, as well as many health professionals working in oncology. It also suggests that Australian health professional and patient perspectives on sex after cancer may no longer be as “mis-matched” as previously reported [23].

There are a number of strengths and limitations of the present study. The strategic nature of our participant sampling did ensure that the perspectives gained reflected experiences of cancer in a broad sense; across types of cancer, relationship status, sexual identity, and patient, partner and professional experience. However, the sample was not ethnically or culturally diverse as most participants were of white-Australian ethnicity. Also, patient and partner participants were, on average, in their middle years. Conclusions cannot be drawn about different constructions amongst such groups, and further research could examine how age and cultural background influence beliefs about sexuality and intimacy after cancer. Equally, all participants volunteered to take part in a study about cancer and sexuality, which meant that they were likely to be comfortable about addressing sex as an issue, or had a particular concern or viewpoint they wished to impart to the research team. Individuals who are not comfortable about discussing sex and intimacy, are very distressed about sexual changes and not able to renegotiate sexual practices, or who do not feel that sex is an important issue to consider in the context of cancer, may express different viewpoints. Other research has demonstrated that many individuals with cancer experience distress as a result of changes to their sexuality and their inability to perform coital sex [4]. Further research on this issue could usefully be undertaken with a randomly selected group of people with cancer, partners, and health professionals. Recognition of the importance of sexuality in the context of cancer and the need for health professionals to take responsibility for initiating discussions around this issue has been emphasized in Australian clinical practice guidelines [71-73]. The findings of this study need to be considered within this context with further research examining experiences in countries where recognition of the importance of sexuality in the context of cancer is not currently reflected in health policy and practice.

## Conclusions

In conclusion, this study has demonstrated the complexity of perspectives about sexuality and intimacy post cancer, which has practical implications for those working in cancer care and survivorship. The constructions of sex and intimacy adopted by health professionals shape their willingness to raise the subject with their patients, as well as the specific information and advice they impart if such discussion is undertaken [23,54], and are therefore worthy of investigation. Constructions of sex also serve to “set the horizon of the possible” in terms of sexual desire and behaviour [74]:16], and influence individuals in their the ability to renegotiate sexual practices in the context of cancer [28,29]. Therapists and other health professionals can play an important role in ameliorating concerns surrounding sexual wellbeing after cancer, by opening and facilitating discussion of sexuality and intimacy amongst couples affected by cancer, as well as providing information that normalizes a wide range of sexual and intimate practices, in order to facilitate such renegotiation. However, it is also important for health professionals to be aware that some individuals consider sexual matters to be a private concern, preferring communication to remain within the couple relationship, and non-sexual intimacy to be more important than sex.

## Competing interests

The authors declare that they have no competing interests.

## Authors’ contributions

JP, JMU and EG devised, planned and coordinated the study. EG was involved in the collection of the Q sort and interview data. JP performed the Q analysis and initial interpretation of the results with JU assisting in the interpretation of the interview data. JP and JMU drafted the manuscript. All authors read, revised and approved the final manuscript.

## Pre-publication history

The pre-publication history for this paper can be accessed here:

http://www.biomedcentral.com/1471-2407/13/270/prepub
